# Evaluation of Rationally Designed Label-free Stem-loop DNA Probe Opening in the Presence of miR-21 by Circular Dichroism and Fluorescence Techniques

**DOI:** 10.1038/s41598-020-60157-5

**Published:** 2020-03-04

**Authors:** Nasrin Farahani, Mehrdad Behmanesh, Bijan Ranjbar

**Affiliations:** 1 Department of Nanobiotechnology, Faculty of Biological Sciences, Tarbiat Modares University, Tehran, Iran; 2 Department of Genetics, Faculty of Biological Sciences, Tarbiat Modares University, Tehran, Iran; 3 Department of Biophysics, Faculty of Biological Sciences, Tarbiat Modares University, Tehran, Iran

**Keywords:** Biosensors, Circular dichroism, Fluorescence spectroscopy, DNA probes, Fluorescent dyes

## Abstract

The characteristic features of stem-loop structured probes make them robust tools to detect targets with high sensitivity and selectivity. The basis of the hairpin based sensors operation is a conformational change that occurs upon hybridization of target with stem-loop probe. The design of the stem-loop probe has an important role in target recognition. Therefore, we designed a label-free stem loop probe for targeting miR-21 as a cancer biomarker investigated by web-based tools; its thermodynamic parameters obtained by thermal UV spectroscopy. The efficiency of stem-loop structure opening in the presence of target and non-target sequences was evaluated by fluorescence spectroscopy and circular dichroism spectro-polarimetry. The results showed that the target sequence opens the structure of hairpin efficiently in comparison to non-target sequences. To optimize the stem-loop hybridization to its target, the buffer ionic strength was changed by adding different concentrations of NaCl, KCl and MgCl_2_. It was shown that buffering conditions have a significant role in loop structure opening and its optimization, led to an increase in sensitivity detection and have improved LOD from 60 pM to 45 pM.

## Introduction

Most DNA or RNA based biosensors use the process of target hybridization with an oligonucleotide probe as the main targeting technology^1^. The sensitivity and selectivity of these biosensors, depend on factors such as oligonucleotide sequence composition, capture probe structure, ionic strength and temperature^2–4^. It is important to consider the ability to mismatch discrimination in designing^5^, which is affected by the structure of the probe. Stem-loop structured probes are useful concept in comparison to other nucleic acid conformations such as linear probes, because the stem-loop probes provide several advantageous aspects in DNA–DNA and DNA–RNA interactions^5,6^. Thermodynamic analysis has shown that in competing reaction between stem-loop formation and target hybridization, higher specificity in target recognition has occurred than discrimination probes that cannot form a stem-loop structure;^5,7^ also there are several studies which distinguished with high mismatch discrimination target from non-target sequences that differ with a single nucleotide^7–10^.

Stem-loop structure is an intramolecular base pairing that can occur in single stranded DNA or RNA if sequences of two regions of the same strand are complementary to each other. In this situation a double-stranded stem forms which has an unpaired loop at ends. This structure is also known as hairpin. If the stem-loop structured oligonucleotide probe properly designed, it would be opened in the presence of sequences complementary to the loop sequence. This feature can be used for real-time measurements and fabrication of a variety of biosensors for detection of unlabeled biomolecules target such as nucleic acids and proteins.

The use of stem-loop structure that can be opened in the presence of specific nucleic acids, were first reported by Tyagi and Kramer as “molecular beacon” (MB) in 1996^11^. MB is a single-stranded DNA oligonucleotide with stem-loop structure that is functionalized with a fluorophore and a quencher at each end. In the absence of the target sequence, a complement for MB loop sequence, the closed stem-loop structure quenches fluorescence. The presence of target leads to the formation of double-stranded DNA structure by opening and hybridization of MB loop sequence with the target sequence, separating the fluorophore and the quencher restores fluorescence^5,12^.

Conventional molecular beacons contain DNA sequences labeled at both ends^13^. Labeling the molecular beacons, faces challenges due to the high cost, limited available probes, rapid photo-bleaching, limited synthesis techniques, and time consuming operation, as well as the possibility of reducing the sensitivity of MB to target^14^. In recent years, researchers have innovated in molecular beacon structures and developed various types of diagnostic sensors and therapeutic methods, based on stem-loop structures, such as label-free MB. Since 1996, the stem loop structures or label-free MB recognized targets have been studied by electrochemistry^15,16^, colorimetric^17,18^ and surface enhanced Raman scattering (SERS)^19,20^ methods.

All biosensors that have a stem-loop structure contain a nucleic acid sequence that captures the target sequence. Designing an appropriate stem-loop structure, is important for the sensitivity and accuracy of detection of capturing the target^21,22^. The specific sequence for efficient hybridization creates dramatic changes in signal responses. An effective stem-loop is a structure that has maximum opening in the presence of the target sequence, and cannot be opened by the non-target sequences. The proper designed stem-loop requires an adequate environment (buffering conditions including buffer type and salts contained in it), incubation time and temperature for hybridization process in such a way that the sensor has the highest performance. So, if we have just a label-free stem-loop DNA sequence, how we can find its thermodynamic properties, investigate the efficiency of its hybridization to target, assess its operation when exposed to the target and non-target agents and evaluate optimum conditions for its performance, with the application of the minimum technical cost and time and before applying it in the final structure of the sensor?

In this study, in response to these questions, we designed a stem-loop structured DNA (capture probe) for targeting miR-21 as a cancer biomarker and used fluorescence spectroscopy and circular dichroism spectropolarimetry techniques as simple and fast techniques for investigation of the stem-loop structure opening in the presence of target and non-target sequences. Among the large number of miRNAs that have been identified, miR-21 was one of the most remarkable targets related to cancers^23^ which its role as an oncogene in targeting tumor suppressor genes such as PTEN, TPM1, and PDCD has been approved^24^. Based on clinical researches, miR-21 is upregulated in various types of cancers. The aberrant expression of miR-21 indicates a failing health, which alert for a malignancy in the body.

Thermodynamic properties of the capture probe were analyzed by thermal UV spectroscopy technique. Since the capture probe was label free, PicoGreen was used as an ultra-sensitive fluorescent nucleic acid stain for quantitating double-stranded oligonucleotide. It was also used to quantify hairpin structure in the presence of target sequence to produce a double-stranded oligonucleotide. The fluorescence intensity will reduce as a result of less amount of double-stranded DNA.

Optimization of the reaction variables was done with fluorescence technique and PicoGreen dye, in order to maximize stem-loop structure opening in the presence of target miR-21 sequence and increase of the detection sensitivity.

## Methods

### Materials and apparatus

All reagents including HEPES and other chemicals for buffer preparation were analytical grade and sourced from Sigma Aldrich. All solutions were prepared using ultrapure Milli-Q water. PicoGreen was purchased from Invitrogen, USA.

Thermal UV-vis absorption spectra were obtained by a Peltier accessory coupled to UV-vis Perkin Elmer Lambda 25 spectrophotometer. Fluorescence measurements were carried out on a Perkin Elmer LS55 fluorescence spectrophotometer and BioTek Cytation 3 Cell Imaging Multi-Mode Reader (USA). CD spectra were recorded on a Jasco J-715 spectropolarimeter (Japan).

### Oligonucleotide synthesis

**Figure 1:**
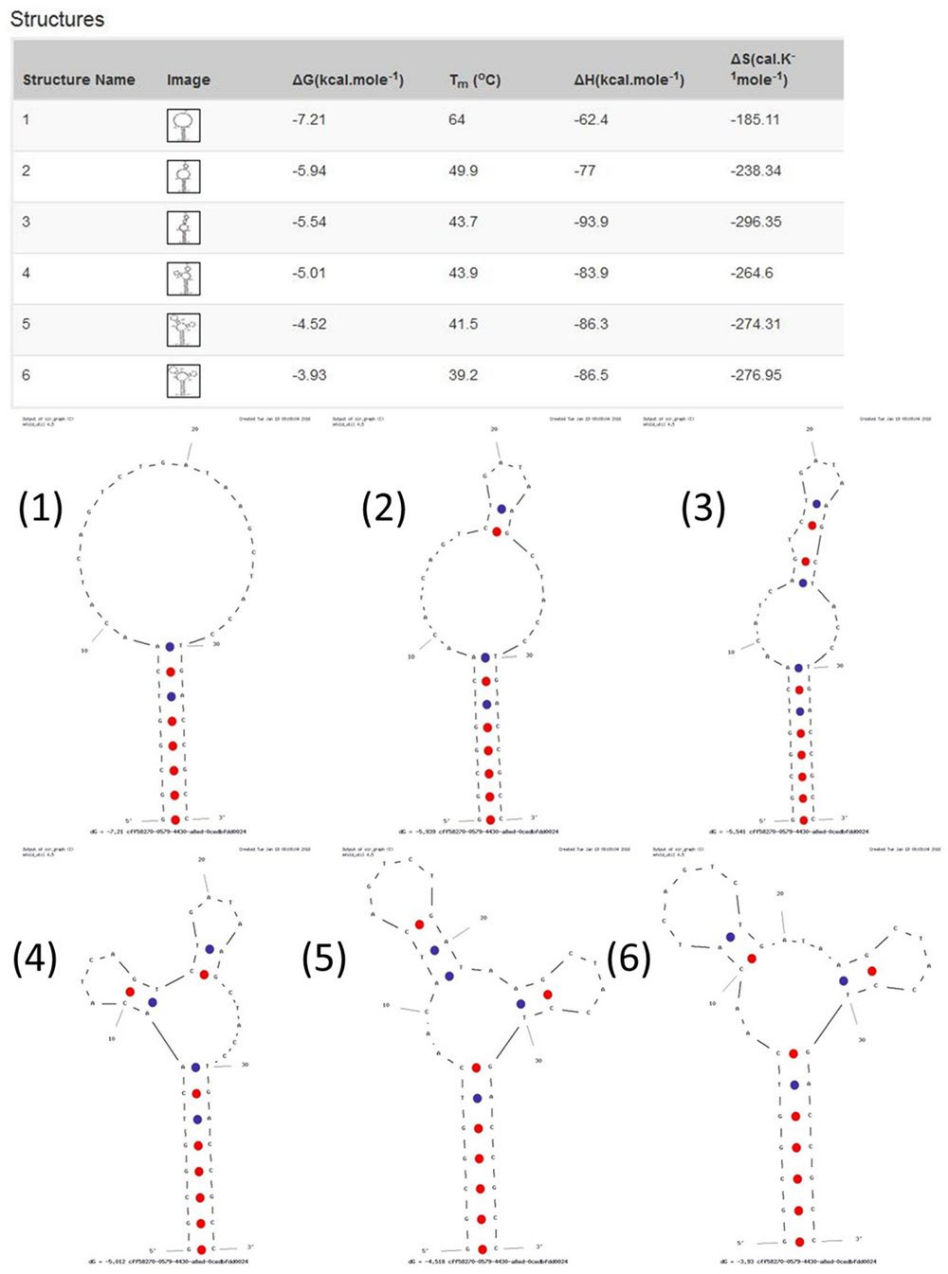
Representation of the secondary structures and thermodynamic properties of capture probe were determined by the Mfold program.

Stem-loop DNA oligonucleotide (capture probe) complementary to the mature nucleotide sequence of miR-21 was designed by web-based tools such as Mfold, OligoAnalyzer and Beacon Designer programs. Prior to capture probe synthesis, the secondary structure and thermodynamic properties of capture probe were determined by the Mfold program^25^ (Fig. 1). Capture probe, cDNA miR-21 (target), one nucleotide mismatch miR-21 and three nucleotide mismatch miR-21 (non-target probes) and sequence that was complementary to miR-21 (Table 1) were synthesized by AnaSpec, Inc. (Canada). Probes were purified by their respective companies by standard desalting and HPLC methods (All probes were gel purified).

**Table 1:** Target and non-target probe sequences.

Name	Abbreviations	Sequence (5′ to 3′)
Stem-loop structured DNA probe	Capture probe	GGC GGT CAA CAT CAG TCT GAT AAG CTA CCT GAC CGC C
cDNA miR-21	miR-21	TAG CTT ATC AGA CTG ATG TTG A
One mismatch cDNA miR-21	mutant1	TAG CTT ATC **G**GA CTG ATG TTG A
Three mismatch cDNA miR-21	mutant3	T**T**G CTT ATC **G**GA CTG AT**C** TTG A
Sequence complementary to miR-21	comp miR	TCA ACA TCA GTC TGA TAA GCT A

Capture probe was formed as 21 nucleotide single-stranded loop and 8 nucleotide GC rich double-stranded stem. Unlike conventional stem-loop that the special-target binding site is located only in the loop area, between short self-complementary stems^22^, in this capture probe part of the stem sequence was also involved in binding to the target miR-21. All synthetic probe sequences were dissolved in Milli-Q water and kept frozen until use.

### Thermal UV-vis studies

Thermodynamic properties of stem loop structure including melting temperature (*T*_*m*_), *ΔG, ΔH* and *∆S* were investigated in a 25 mM HEPES buffer (pH 7.4). For this purpose, 1 µM of the capture probe was prepared in HEPES buffer. In order to form stem-loop and avoid creating dipolymer, the capture probe was then treated at 75 °C for 30 min and ice-cold water for 10 min^26^. Absorbance of the sample was measured at 260 nm with thermal UV-vis spectroscopy from 25 °C to 85 °C. (1 min intervals at each degree for 3 times). Calculation of the thermodynamic properties is shown in the Supporting Information, Section 1.

### Fluorescence spectroscopy studies

Opening Stem-loop structure of capture probe in the presence of the target miR-21 and non-target mutant 1 and mutant 3 was studied by PicoGreen fluorescence assay in LS55 spectrofluorimeter. PicoGreen dye exhibits a fluorescence emission upon binding to double-stranded DNA to detect and quantify it^27^. So, if the capture probe be opened in the presence of target or non-target probes, a double-stranded structure will be formed and fluorescence emission will be generated. Fluorescence intensity alterations are directly related to the amount of double-stranded DNAs. Samples including capture probe, miR-21, mutant 1, mutant 3, miR-comp miR pair, capture-miR21 pair, capture-mutant1 pair and capture-mutant 3 pair were prepared (0.05 µM final concentrations in 25 mM HEPES (pH 7.4)). In the following, all of the capture probes were initially prepared as described before, and then the miR-21 and mutants were added to them and incubated for 2 h at 0 °C, 25 °C, 37 °C and 45 °C. Finally, all samples were treated with PicoGreen stain and were incubated for 5 min. For fluorescence measurement, excitation was performed at 480 nm and emission spectra were recorded between 500–550 nm. Each reading was done more than 4 times.

### CD studies

Conformational changes of capture probe structure in the presence of miR-21, mutants and the confirmation of loop formation were studied by CD spectroscopy in the UV region (200–300 nm)^28^. Smoothing and analyzing data was done by J-715 Jasco data analyzer software. All samples were prepared as stated in the previous section but the final concentration of the samples was 20 μM and also PicoGreen dye was not used. Each assay was performed more than 4 times.

### Optimization of hybridization process

In order to maximize opening of the capture probe in the presence of miR-21, hybridization process in various buffering conditions (including different concentrations of Na^+^, K^+^ and Mg^2+^ ions) was investigated by fluorescence technique.

We designed experiments to determine the appropriate levels of NaCl, KCl and MgCl_2_. For this purpose, different concentrations (0 to 1 M) of each salt was prepared in 25 mM HEPES buffer and capture probe was added to 0.05 µM final concentration. After incubation at 75 °C for 30 min and ice-cold water for 10 min, miR-21 in 0.05 µM final concentration was added to each reaction and samples were incubated for 2 h at 25 °C. Finally, PicoGreen was applied and the fluorescence emission was recorded at 530 nm. Four samples which had the highest rate of hybridization compared to the control, were selected and the proper levels of each salt were obtained. Levels of temperature and time variables are selected by inspecting practical observation.

## Results and Discussion

### Thermal UV–visible spectroscopic measurements

To increase the efficiency of the biosensors based on the hairpin structures in detecting the target oligonucleotides, a stem-loop DNA structure (capture probe) was designed for targeting miR-21, which is a valuable marker for a number of diseases such as several cancers. In this study we have considered all the points mentioned by other researchers^22,29^ who had worked on designing stem-loop and MB structures. The design of capture probe was in such a way that it would have the highest thermodynamic stability in hairpin conformation while it will be open in the presence of miR-21 at the same time. In solution, a temperature dependent equilibrium between hairpin and random coil conformations has established^29^. The temperature in which half of the oligonucleotides are in the stem-loop conformation, is called melting temperature (*T*_*m*_). Therefore, finding the *T*_*m*_ and the temperature range in which most structures are in the form of stem-loop, is necessary for the sensor function.

**Figure 2:**
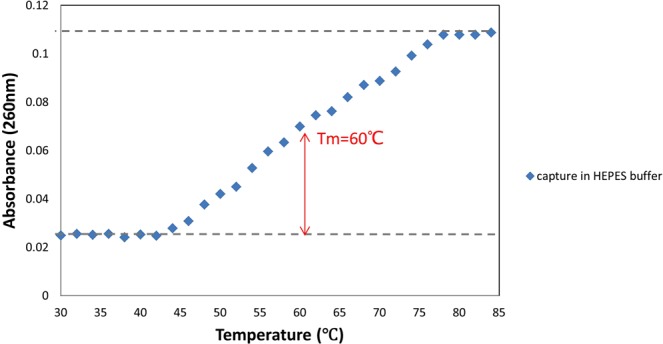
UV spectra of the stem-loop DNA at 260 nm as a function of temperature.

To characterize the thermodynamic properties of designed stem-loop structure, melting curve assay of capture probe was executed (Fig. 2). As seen in Fig. 2, as the temperature increased, the conformation of capture probe was changed from stem-loop to random-coil and as a result, the absorbance in 260 nm was gradually increased. *T*_*m*_ value was derived from the melting curve and other thermodynamic properties were obtained (see Table S-1). At temperatures below 60 °C, capture probe was in the hairpin conformation, so it was the appropriate temperature range for working with this sensor.

The *T*_*m*_ value obtained by thermal experiments was compared to *T*_*m*_ estimated with DNA mfold program expanded by Michael Zuker^30^ (available at http://unafold.rna.albany.edu/q=mfold/DNA-Folding-Form). The observed *T*_*m*_ (60 °C) for the capture probe was about four degrees lower than the predicted *T*_*m*_ (64 °C), which could be due to the difference in buffer and salts. The *T*_*m*_ was predicted in the presence of 1 M NaCl concentration while thermal experiment was carried out in 25 mM HEPES buffer without any salt addition. The difference between the predicted and observed *T*_*m*_ was also reported previously^22^. The surrounding environment around DNA is very effective in its hybridization process. The effect of different concentrations of NaCl on the *T*_*m*_ of the hairpin was studied^29^, and it was shown that positively charged Na^+^ ions had a stabilizing effect on the hybridized DNA strands in solution so that, by increasing salt concentration, the *T*_*m*_ of the hairpin increased. Therefore, this can be a reason for the higher predicted *T*_*m*_ than the observed *T*_*m*_.

### Investigation of Stem-loop structure opening in the presence of target and non-target markers

In sensors based on the stem-loop structure, it is necessary to precisely design the structure of the hairpin in order to identify the target marker with high sensitivity and selectivity. Therefore, the proper design of the hairpin plays an important role in the function of this category of sensors. In order to ensure the design of the hairpin structure is appropriate, its opening in the presence of the target marker should be approved and if the structure was not suitable, a new structure should be redesigned. All researchers have used dye and quencher modified structures in the form of MB, to verify the efficiency of the hairpin structures. For the first time, we assessed the opening of the hairpin structure without using dye-quencher modification, and we used CD and fluorescence techniques for quantification the changes.

### Fluorescence spectroscopy

**Figure 3:**
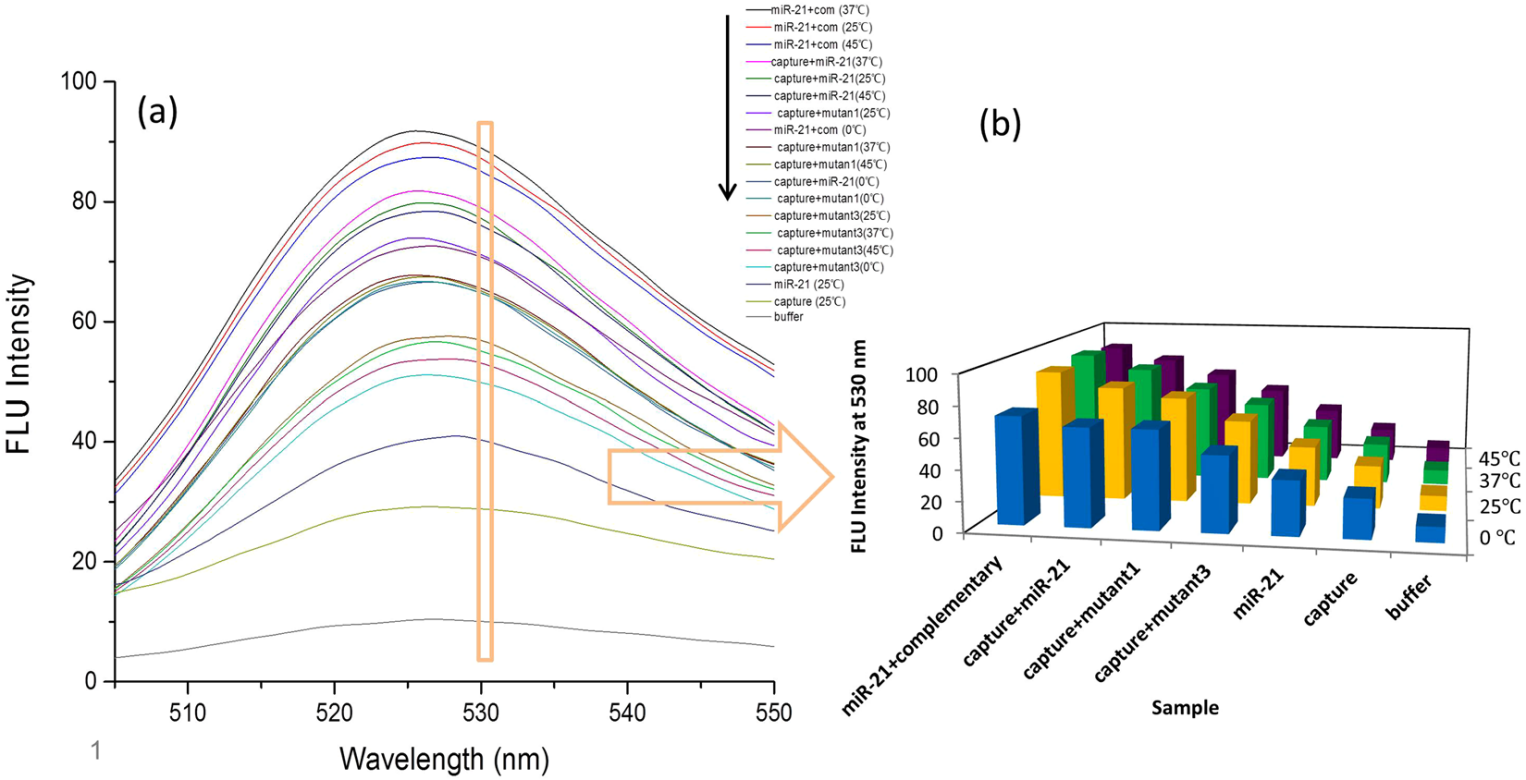
Fluorescence intensity of different samples under various temperature conditions.

PicoGreen is a fluorescent dye that its emission enhances in the presence of double stranded DNA. PicoGreen does not show DNA base preference and provides a sensitive and reliable technique for the quantitation of dsDNA^31^. Stem-loop structured sensors are designed as hairpin (capture probe) structures to identify the target marker with high sensitivity and selectivity. In this study, we used PicoGreen to quantify the capture probe opening in the presence of miR-21 or mutants markers. Figure 3 shows the highest fluorescence intensity for miR21-comp miR pair samples at 37 °C, 25 °C and 45 °C, respectively, indicating the highest rate of hybridization. This can be detected by higher fluorescence intensity due to the increase in the number of complementary base pairs^32^. These assays were standard for comparison with other samples. After standard samples, the highest fluorescence intensity was related to the capture-miR21 pair samples at 37 °C, 25 °C and 45 °C, indicating opening of the capture stem-loop structure in the presence of the miR-21 marker. The best temperatures for hybridization were 37 °C and 25 °C. Increasing the temperature to 45 °C or decreasing to 0 °C reduced binding of the target marker to the capture. Interestingly, it was seen that this structure had the ability to distinguish the target marker from one or three mismatch mutants in the range of 25 °C to 45 °C.

The fluorescence intensity of the miR-21, mutant 1 and mutant 3 samples was similar at the same temperature. Also, the change in incubation temperature had a small effect on the fluorescence intensity of the samples and the highest fluorescence was observed for all three samples at 25 °C and 37 °C. In order to avoid drawing a large number of charts, only miR-21 sample was displayed at 25 °C. It should be noted that the reason for the observation of fluorescence in these samples was the creation of about 8–10 base pairs nucleotide self-dimers. The fluorescence intensity of the capture probe sample also changed very slightly with increasing incubation temperature, and therefore only a capture chart was displayed at 25 °C. It is noteworthy that capture in a stem-loop conformation with eight nucleotide double stranded stem and the fluorescence was due to the stem. Capture fluorescence intensity was less than miR-21 and mutants samples, which show stem-loop formation. In the absence of loop formation but with creation of self-dimer structures, with increasing in number of nucleotide pairs, the fluorescence intensity was higher than miR-21 sample. This issue was examined and verified by investigating the intensity of fluorescence in both loop and lack of loop formation (The results are not listed here).

The capture probe-mutant1 hybridization observed at 25 °C was the highest, then 37 °C and 45 °C, respectively. For the capture probe-mutant 3 hybridization, best results were obtained at 25 °C. It seems that the coupling between the mutant 1 and mutant 3 with capture probe was better at 25 °C than 37 °C. It could be due to the lack of complete pairing and also decrease of connections’ tightness by increasing temperature. For all samples which incubated at 45 °C, the fluorescence intensity had decreased. This intensity reduction can either be due to a decrease in the rate of marker hybridization to the capture probe or high temperature effect on fluorescence, that is, fluorescence decreases with temperature due to competing process of internal conversion which reduces the number of molecules which comes down to ground state from excited state by radiative transition. Even considering the possibility of an internal conversion process, the low fluorescence intensity of capture probe-mutant samples relative to the capture probe-miR21, indicates a reduction in the rate of hybridization at this temperature. In all samples, the lowest fluorescence intensity was related to incubation at 0 °C (see also Figs. S-2 and S-3).

Figure 3 showed the highest fluorescence intensity of capture-miR21 pair, observed at 37 °C, indicating a better hybridization binding of the miR-21 targeting marker to the capture probe. In general, by increasing the temperature, the potency of the stem-loop structure to targeting miR-21 has increased, but the increasing temperature to 45 °C has reduced the amount of hybridization, owing to the weakness of the bonds between loop and target marker. So, the temperature of 45 °C or more, due to its proximity to the melting temperature of capture-miR21 pair, reduces the rate of hybridization.

The fluorescence assay was done to investigate the efficiency and suitability of this technique for evaluating the stem-loop structure. A proper stem-loop structure has the ability to detect single nucleotide variations in the target sequence^7,33^. As the results of this assay, the use of the fluorescence technique with using PicoGreen dye, greatly has demonstrated the ability of the hairpin structure to differentiate sequences with even one nucleotide mismatch. Therefore, it was a good technique to assess the design of the stem-loop structure without the need for using dye and quencher labels.

### CD spectropolarimetry

**Figure 4:**
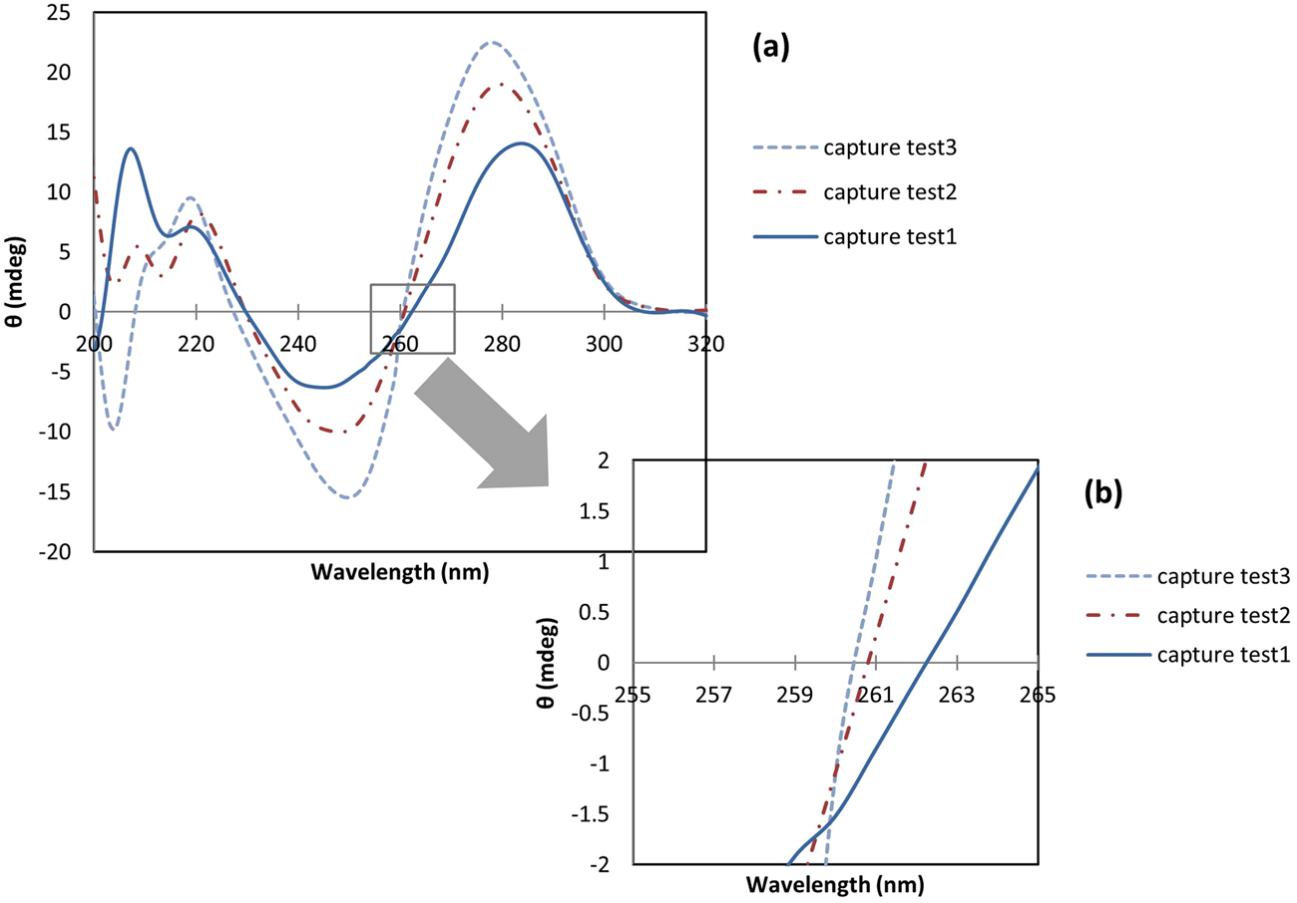
Circular dichroism spectra above 200 nm for different conformational forms of capture probe (**a**). A shift of the long-wavelength crossover and increase in CD signal intensity at 260 nm as result of denaturation (**b**).

CD spectroscopy is a valuable tool for searching conformational isomerization of DNA^34^. This technique is sensitive to the asymmetry of a molecule, and is used to determine the secondary structure of nucleic acids^35^. In the current study it was used to assess the secondary structure alterations of the capture probe in the presence of miR-21 or mutants. Three samples containing capture probe were prepared to confirm loop formation. The samples were heated at 75 °C for 30 minutes. CD spectrum of the first sample (test 1) was measured directly after heating and no cooling. The second sample (test 2) was immediately placed in ice water after heating, to form a stem-loop structure, and the third sample (test 3) gradually returned to room temperature over time. It is expected that in test 3 sample, in addition to the stem-loop structure, other structures including self-dimer, would be generated. CD spectra of these two samples were also measured. Figure 4(a) indicates that all samples show a positive band around 278 nm and a negative band around 245 nm. CD spectrum of test 3 sample had the characteristics of the B-DNA. Most DNA oligonucleotides prefer B-form in neutral aqueous buffers at moderate salt^36^.The B-DNA has a maximum at 275 nm and a minimum at 245 nm which are approximately equal^28^, although the position and amplitudes of the CD bands vary considerably depending on the sequence^34^. In test 2 sample, both negative and positive CD signal intensity decreased. Such variations in amplitudes can be related to conformational changes in oligonucleotides^34,37^. In self-dimer formation, 20 nucleotides are hybridized from two different capture probes and produce almost two-stranded structure, but in the case of loop formation, the two ends of a capture probe are connected together and create a double-stranded 8-nucleotide stem and a single-stranded loop. Therefore, decreasing in CD signals intensity of the second sample compared to the third sample can be due to the formation of stem-loop structure with a lower percentage of double stranded structures than the self-dimer. Duplex to hairpin transition is usually associated with reduction of CD signal intensity due to single stranded loop^34^. Among these three samples, first sample had the lowest CD signal intensity. Heating of the first sample without cooling led to the formation of a single-stranded oligonucleotide structure. In thermal denaturation of DNA, the magnitudes of the major CD bands above 230 nm decrease as the temperature increases because of unstacking of the bases with diminish adjacent bases interaction^38^. As seen in Fig. 4(b), a shift in long-wavelength crossover from 261 nm to 262 nm and an increase in CD signal intensity at 260 nm occurred which can be an indicator of denaturation^38^.

**Figure 5:**
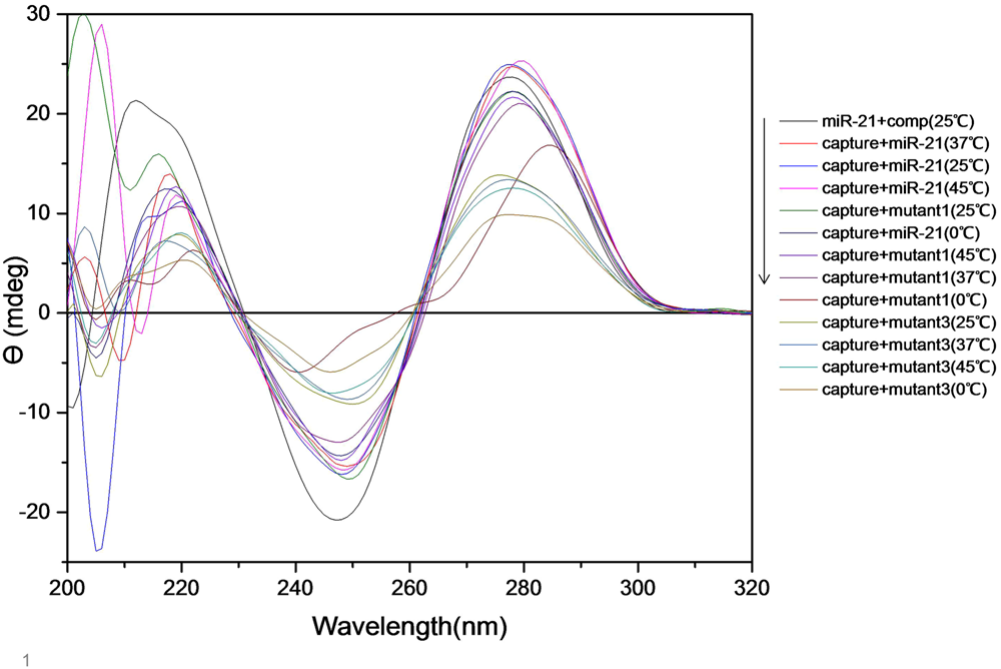
CD spectra of different samples under various temperature conditions.

Similar to the results obtained from fluorescence measurements, there was no meaningful difference between the miR-21, mutant 1 and mutant 3 spectra with the change in incubation temperature. In order to prevent the use of a large number of spectra in the graphs, only the miR-21 spectrum has displayed at 25 °C. Also, the looped capture probe spectrum did not change significantly with the incubation temperature (the spectrum is only shown at 25 °C (Fig. 5)).

**Figure 6:**
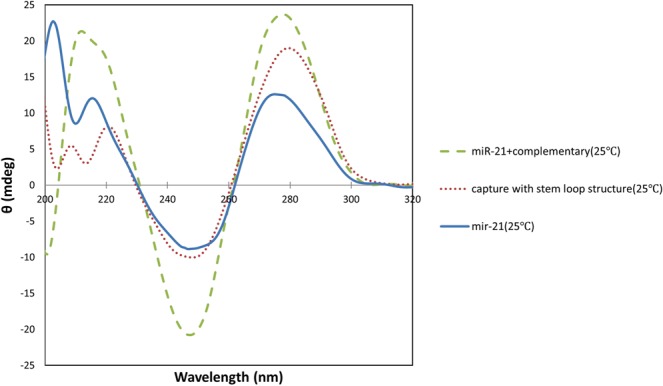
Comparison of miR21-comp miR pair, stem-loop capture probe and miR-21 CD spectra at 25 °C.

The miR21-comp miR pair spectrum had a positive band at 278 nm and a negative band at 245 nm with almost equal intensity (2–3 nm difference) that was a feature indicative of B-form. This spectrum was used as a template for the formation of duplex. So that, in case of capture probe hybridization to each of the markers, the best results were from a sample that the spectrum was most similar to the template. The miR-21 also had a positive band about 278 nm and a negative band about 245 nm, but the intensity of these bands were less than the miR21-comp miR pair, which represented the lower content of the secondary structures in the miR-21 (see Fig. 6).

As shown in Fig. 5, the highest rate of hybridization was related to capture-miR21 pair at 37 °C, 25 °C and 45 °C respectively. Also, the lowest intensities were relevant to capture-mutant3 pair at all incubation temperatures. In order to better analyze the data, the samples were drawn on separate charts (Figs. S-4 and S-5). As the results show, CD technique has less sensitivity than fluorescence technique to show capture binding to markers at different temperatures. So that, changing in the incubation temperature of the samples, with the exception of the temperature of 0 °C, did not lead to a considerable change in the CD spectrum.

At all temperatures, the CD signal intensity of capture-miR21 pair was more than other samples, which can indicate the formation of a larger number of B-form double-stranded DNA structures in these samples than in others. The difference in the CD magnitude between capture-miR21 and capture-mutant1 was less than capture-mutant3, indicating a lower rate of hybridization in the capture-mutant3 samples. According to the results obtained from this assay, the temperature of 37 °C was the best for incubation, since only in this temperature the difference in CD magnitude in both the positive and negative bands was observed for capture-miR21 as compared to the other two mutant markers, as well as the highest rate of capture-miR21 hybridization occurred in this temperature. The CD technique did not have a good ability to compare the capture probe hybridization to each of the markers at different temperatures, but it could ideally distinguish between the capture probe binding to the miR-21 and the mutants at all temperatures. The results of this assay corresponded to the results obtained by the fluorescence method.

### Optimization of capture probe opening in the presence of miR-21

Stem-loop structures have shown higher sensitivity and specificity in target discrimination in comparison to linear probes. Most studies on DNA hybridization have been performed in aquatic environments, so the buffer composition plays a very important role in hybridization efficiency and performance^39^. In addition, we seek the best sensor response to the target at the minimum temperature and time. It has been reported that increasing the salt concentration in the buffer leads to a faster hybridization rate^39^. In this study, in order to increase the efficiency of the capture probe which had a stem-loop structure, in targeting miR-21, we changed the buffer ionic strength by adding different concentrations of NaCl, KCl and MgCl_2_ and investigated their effects on the hybridization process.

### Determination of salts levels

**Figure 7:**
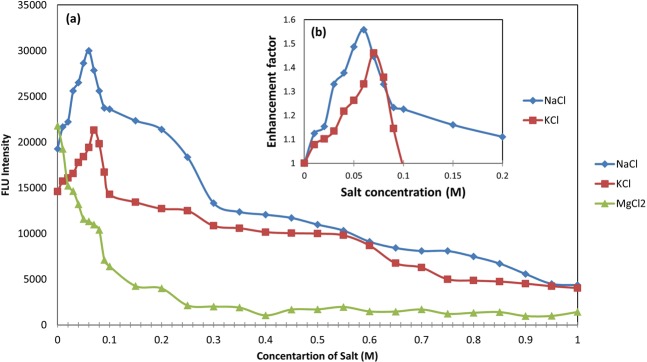
Alteration of fluorescence intensity at 530 nm as a result of the addition of NaCl, KCl and MgCl_2_ from a concentration of 0 to 1 M (**a**). Fluorescence enhancement factor for concentrations of 0 to 0.2 M NaCl and 0 to 0.1 M KCl (**b**). The fluorescence enhancement factor of the reference sample (salt-free) is 1, and samples with a fluorescence intensity higher than the reference have a fluorescence factor more than 1.

The effects of the salt concentrations on the hybridization are shown in Fig. 7(a). Hybridization was performed at 25 mM HEPES buffer with different concentrations (0.01–1 M) of each salt.

It can be seen that in samples containing NaCl, the fluorescence intensity increased with increasing in salt concentration up to 0.2 M, as compared to the salt-free sample (reference sample containing 0 M NaCl). When the NaCl concentration was further increased from 0.2 M to1 M, the fluorescence intensity of capture-miR21 pair was decreased. As shown in Fig. 7(b), as the NaCl concentration was increased from 0 M to 0.06 M, the fluorescence enhancement factor that is calculated by the ratio of the fluorescence intensity of the sample containing salt to salt-free sample^3^, increased from 1 (implying no added salt) to 1.5, and the enhancement factor decreased to 1.1 upon further increasing the NaCl concentration to 0.2 M. Accordingly, the highest fluorescence enhancement factor was related to a concentration of 0.06 M NaCl and in the following, 0.04 M, 0.05 M and 0.07 M concentrations have it so, respectively.

The effect of adding various concentrations of KCl on the capture probe-miR21 hybridization is shown in Fig. 7(a). By increasing the concentration of KCl from 0 M to 0.09 M, the fluorescence intensity increased compared to the reference sample, but the rate of this increase was different for various concentrations, as increasing the concentration from 0 M to 0.07 M, increased the fluorescence enhancement factor from 1 to 1.46 and a further increase in the concentrations to 0.09 M, decreased the enhancement factor to 1.14 (see Fig. 7(b)). As a result, the best concentration for KCl was 0.07 M, 0.08 M, 0.06 M and 0.05 M, respectively.

The study on the effect of MgCl_2_ on hybridization showed a significant decrease in fluorescence intensity at all concentrations (Fig. 7(a)).

Negatively charged phosphates on the backbone of nucleic acid molecules, gives them poly-anionic nature. The charged cations, such as sodium, potassium and magnesium ions can neutralize these negative charges and help nucleic acid molecules folding into the compact native structures by reducing the repulsion between phosphate residues^4^. The hybridization of stem- loop structures with targets are also significantly affected by the concentration of salts^39^. It has been demonstrated that increasing the concentration of NaCl from 0 mM to 1000 mM, increases the melting temperature of the hairpin and so, Na^+^ ions has a stabilizing effect in the solution on the hybridized DNA strands^29^. An increase in the pairing reaction of the complementary ends of the beacon with increasing NaCl concentration have also been reported^2^. Increasing the stability of the stem-loop structure to the extent that it can be opened in the presence of the target, leads to a better distinction between the target and mutants, as a result of the hairpin-target hybridization becomes more favorable thermodynamically and kinetically. Since factors such as the length of strands, their base composition and system temperature determine the Na^+^ concentration needed for the coupling of complementary oligonucleotides^2^, we observed the best rate of capture probe-miR21 hybridization at 25 °C at a concentration of 0.06 M NaCl, in this study. Reducing the rate of hybridization in higher NaCl concentrations can be due to enrichment of the capture probe and target with cations, because the accumulation of positive charges on the chains would create repulsion again and retard the hybridization process^3^. Adding KCl had similar effects to NaCl, on capture probe-target hybridization. However, in similar concentrations, KCl had less fluorescence enhancement factor than NaCl. This can be explained by the fact that smaller cations are more effective in stabilizing the double-stranded DNA, because they get closer to the phosphates and grooves and consequently create stronger interactions with the double-stranded DNA^4^.

We have found one reason for decreasing of fluorescence intensity in the presence of MgCl_2_. Since other researchers have reported an increase in the hybridization of complementary sequences, as well as beacon and target in the presence of certain concentrations of MgCl_2_, it seems that the difference in the results of our experiment with others can be due to the sensitivity of PicoGreen dye to divalent cations^27^. Therefore, the use of PicoGreen dye may not be a suitable method for investigating the hybridization process in the presence of MgCl_2_.

**Figure 8:**
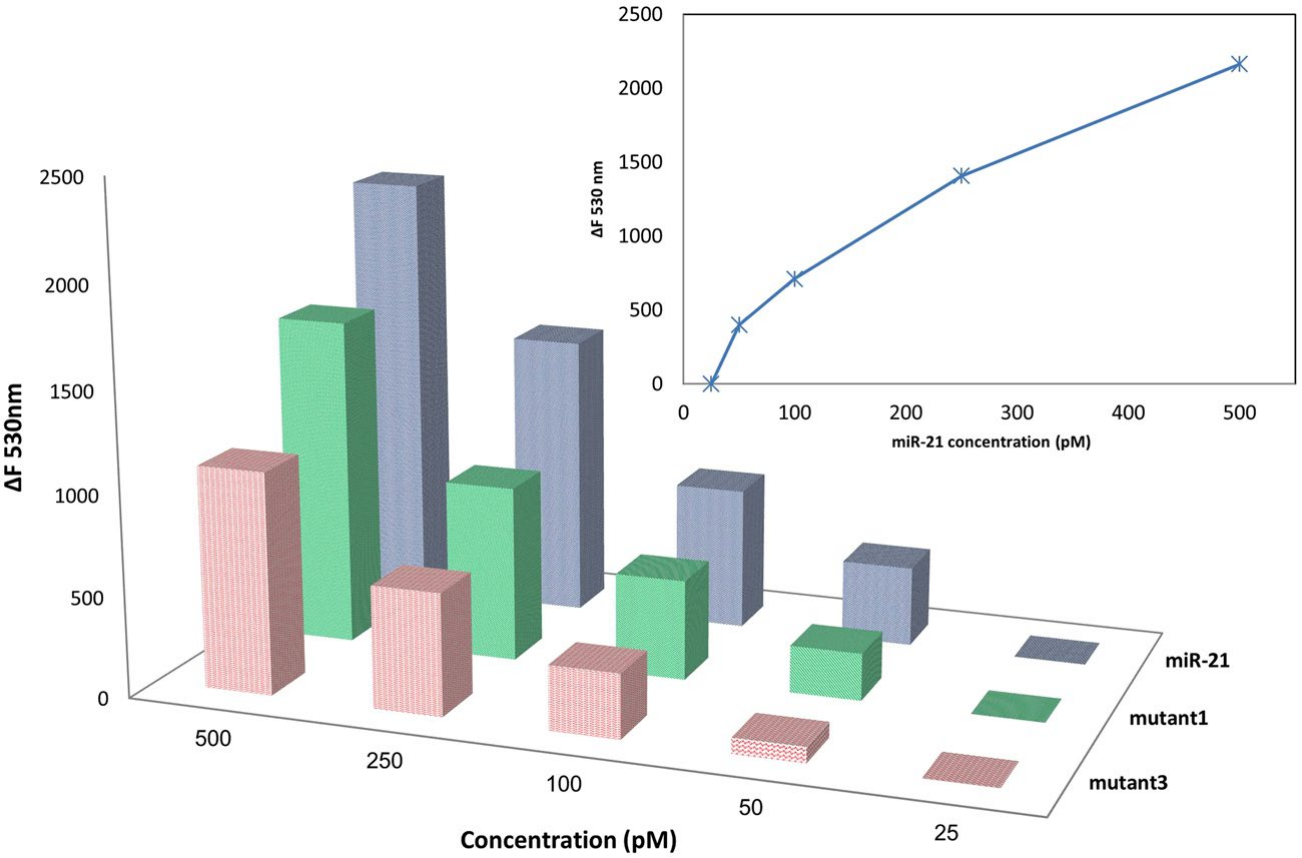
Sensitivity assay of stem-loop capture probe in the presence of different concentrations of target (miR-21) and non-target (mutant 1 and mutant 3) oligonucleotides by fluorescence method. ΔF530 nm = (fluorescence intensity of capture probe in the presence of marker - fluorescence intensity of marker).

Finally, sensitivity of the stem-loop capture probe in identifying miR-21 and differentiation of the mutants was measured in HEPES 25 mM (3 h, 37 °C) in the presence of different concentrations (500 pM-25 pM) of miR-21, mutant 1 and mutant 3, using the fluorescence method. As shown in Fig. 8, sensitivity of this probe was around the pico molar range, and the limit of detection (LOD) was 60 pM that was improved to 45 pM using optimal working conditions (see Table 2).

**Table 2:** Summary of the optimal working conditions.

	Factors	Level Desc.
**1**	NaCl	0.06
**2**	KCl	0.07
**3**	MgCl_2_	0.00
**4**	Time	3 h
**5**	Temperature	37 °C

## Conclusions

In conclusion, we have designed and characterized a single stranded DNA oligonucleotide sequence with a stem-loop structure for miR-21 detection. Sensors based on hairpin structures, such as molecular beacons are very suitable for microRNA identification because they are able to detect short length and relatively low abundance microRNAs with a differentiation of even single nucleotide mismatches. As the first time, the capability of hairpin probe to distinguish the target marker from other non-target markers were successfully investigated by means of CD and fluorescence techniques, without using dye and quencher modifications. Finally, the change in the rate of capture probe-miR21 hybridization in the presence of various ionic conditions was assessed and the optimal level for each of the salts, as well as other parameters including incubation temperature and time were determined. The results of this study clearly showed that CD and fluorescence were appropriate techniques for evaluation of rationally designed structured probes such as hairpin probes hybridization efficiency to target and non-target sequences. Therefore, without the need of using of labeled sequences and only using the simple and fast techniques, we can design or select an optimized stem-loop structure for fabricating hairpin based sensors.

## Supplementary information

Supporting Information.

## References

[1] Gao Y, Wolf LK, Georgiadis RM (2006). Secondary structure effects on DNA hybridization kinetics: a solution versus surface comparison. Nucleic Acids Res..

[2] Markarian MZ, Schlenoff JB (2010). Effect of molecular crowding and ionic strength on the isothermal hybridization of oligonucleotides. J. Phys. Chem. B.

[3] Yao G, Tan W (2004). Molecular-beacon-based array for sensitive DNA analysis. Anal. Biochem..

[4] Tan Z-J, Chen S-J (2006). Nucleic Acid Helix Stability: Effects of Salt Concentration, Cation Valence and Size, and Chain Length. Biophys. J..

[5] Broude NE (2002). Stem-loop oligonucleotides: A robust tool for molecular biology and biotechnology. Trends Biotechnol..

[6] Bockisch B, Grunwald T, Spillner E, Bredehorst R (2005). Immobilized stem-loop structured probes as conformational switches for enzymatic detection of microbial 16S rRNA. Nucleic Acids Res..

[7] Bonnet G, Tyagi S, Libchaber A, Kramer FR (1999). Thermodynamic basis of the enhanced specificity of structured DNA probes. Proc. Natl. Acad. Sci. USA.

[8] Crey-desbiolles, C., Ahn, D. & Leumann, C. J. Molecular beacons with a homo-DNA stem: improving target selectivity. *Nucleic Acids Res*. **33**, (2005). 10.1093/nar/gni076 PMC1090445 15879349

[9] Mhlanga MM, Malmberg L (2001). Using Molecular Beacons to Detect Single-Nucleotide Polymorphisms with Real-Time PCR. Methods..

[10] Tsourkas A, Behlke MA, Bao G (2002). Hybridization of 2 ¢ - O -methyl and 2 ¢ -deoxy molecular beacons to RNA and DNA targets. Nucleic Acids Res..

[11] Tyagi S, Kramer FR (1996). Molecular Beacons: Probes that Fluoresce upon Hybridization. Nat. Biotechnol..

[12] Giannetti A (2015). Optical fiber nanotips coated with molecular beacons for DNA detection. Sensors (Switzerland).

[13] Tan X, Wang Y, Armitage BA, Bruchez MP (2014). Label-free Molecular Beacons for Biomolecular Detection. Anal. Chem..

[14] Zheng J (2015). Rationally designed molecular beacons for bioanalytical and biomedical applications. Chem. Soc. Rev..

[15] Li Xiaolu, Guo Jing, Zhai Qian, Xia Jing, Yi Gang (2016). Ultrasensitive electrochemical biosensor for specific detection of DNA based on molecular beacon mediated circular strand displacement polymerization and hyperbranched rolling circle amplification. Analytica Chimica Acta.

[16] Miao X, Guo X, Xiao Z, Ling L (2014). Electrochemical molecular beacon biosensor for sequence-specific recognition of double-stranded DNA. Biosens. Bioelectron..

[17] Hu D, Huang Z, Pu F, Ren J, Qu X (2011). A Label-Free, Quadruplex-Based Functional Molecular Beacon (LFG4-MB) for Fluorescence Turn-On Detection of DNA and Nuclease. Chem. Eur. J..

[18] Zhang L, Zhu J, Li T, Wang E (2011). Bifunctional Colorimetric Oligonucleotide Probe Based on. Anal. Chem..

[19] Li Y (2016). A Nanostructured SERS Switch Based on Molecular Beacon-Controlled Assembly of Gold Nanoparticles. nanomaterials.

[20] Papadopoulou E, Bell SEJ (2011). DNA reorientation on Au nanoparticles: label-free detection of hybridization by surface enhanced Raman spectroscopy. Chem. Commun.

[21] Conde, J., Rosa, J. & Baptista, P. Gold-Nanobeacons as a theranostic system for the detection and inhibition of specific genes. *Protoc. Exch*. 1–35 10.1038/protex.2013.088 (2013).

[22] Tsourkas A, Behlke MA, Rose SD, Bao G (2003). Hybridization kinetics and thermodynamics of molecular beacons. Nucleic Acids Res..

[23] Zhou X (2014). Prognostic value of miR-21 in various cancers: An updating meta-analysis. PLoS One.

[24] Toiyama Y (2013). Serum miR-21 as a diagnostic and prognostic biomarker in colorectal cancer. J. Natl. Cancer Inst..

[25] Baker MB, Bao G, Searles CD (2012). *In vitro* quantification of specific microRNA using molecular beacons. Nucleic Acids Res..

[26] Yin H, Zhou Y, Zhang H, Meng X, Ai S (2012). Electrochemical determination of microRNA-21 based on graphene, LNA integrated molecular beacon, AuNPs and biotin multifunctional bio bar codes and enzymatic assay system. Biosens. Bioelectron..

[27] Singer VL, Jones LJ, Yue ST, Haugland RP (1997). Characterization of PicoGreen Reagent and Development of a Fluorescence-Based Solution Assay for Double-Stranded DNA Quantitation. Anal. Biochem..

[28] Ranjbar B, Gill P (2009). Circular dichroism techniques: Biomolecular and nanostructural analyses- A review. Chem. Biol. Drug Des..

[29] Jonstrup AThyssen, Fredsoe J, Andersen AH (2013). angaard. DNA hairpins as temperature switches, thermometers and ionic detectors. Sensors (Basel)..

[30] Zuker M (2000). Calculating nucleic acid secondary structure. Curr. Opin. Struct. Biol..

[31] Georgiou CD, Papapostolou I (2006). Assay for the quantification of intact/fragmented genomic DNA. Anal. Biochem..

[32] Azizi A, Ranjbar B, Moghadam TT, Bagheri Z, Baglou SR (2014). Surface plasmon resonance coupled circular dichroism of DNA–gold nanorods assembly. J. Phys. D. Appl. Phys..

[33] Tyagi S, Bratu DP, Kramer FR (1998). Multicolor molecular beacons for allele discrimination. Nat. Biotechnol..

[34] Kypr J, Kejnovská I, Renčiuk D, Vorlíčková M (2009). Circular dichroism and conformational polymorphism of DNA. Nucleic Acids Res..

[35] Sprecher CA, Baase WA, Johnson WC (1979). Conformation and circular dichroism of DNA. Biopolymers..

[36] Fasman, G. D. *Circular Dichroism and the Conformational Analysis of Biomolecules; Climate Change 2013 - The Physical Science Basis* **1**, (Springer, 1996).

[37] Vorlickova M, Kejnovska I, Bednarova K, Renciuk D, Kypr J (2012). Circular dichroism spectroscopy of DNA: from duplexes to quadruplexes. Chirality..

[38] Gray DM, Ratliff RL, Vaughan MR (1992). Circular dichroism spectroscopy of DNA. Methods Enzymol..

[39] Yang, C. J. & Tan, W. *Molecular Beacons;* (Springer, 2013).

