# Supplementary material for: Evaluation of Rationally Designed Label-free Stem-loop DNA Probe Opening in the Presence of miR-21 by Circular Dichroism and Fluorescence Techniques
Source: Sci Rep. 2020 Mar 4;10:4018. doi: 10.1038/s41598-020-60157-5 (PMC7055349; doi:10.1038/s41598-020-60157-5)
Supplement: Supplementary file 1 — Supporting Information. [file 41598_2020_60157_MOESM1_ESM.docx]

Supporting Information

**Evaluation of Rationally Designed Label-free Stem-loop DNA Probe Opening in the Presence of miR-21 by Circular Dichroism and Fluorescence Techniques**

**Nasrin Farahani^1^, Mehrdad Behmanesh^2^, Bijan Ranjbar^1,3*^**

^1^ Department of Nanobiotechnology, Faculty of Biological Sciences, Tarbiat Modares University, Tehran, Iran

^2^ Department of Genetics, Faculty of Biological Sciences, Tarbiat Modares University, Tehran, Iran

^3^ Department of Biophysics, Faculty of Biological Sciences, Tarbiat Modares University, Tehran, Iran

* Corresponding author: E-mail: ranjbarb@modares.ac.ir

**1- Characterization of thermodynamic properties of Capture probe**

The Dissociation constant (K_s→r_) of the capture probe characterizing the transition between stem-loop conformation (s) and random coil (r), is described by the equation [1]:

where A_obs_ is the absorbance intensity in 260 nm at a given temperature, A_N_ is the absorbance intensity of the capture probe in the form of stem-loop (measured at 15°C), and A_D_ is the absorbance intensity of the capture probe in the form of random coil (measured at 80°C) ^1^.

Free energy$\left(\Delta G_{s\to r}^{o}\right)$, changes in enthalpy$(\Delta H_{s\to r}^{o})$ and entropy $(\Delta S_{s\to r}^{o})$ of the system were obtained by equation [3]:

$$\Delta G_{s\to r}^{o}=-R\theta\ln K_{s\to r}(\theta)=\Delta H_{s\to r}^{o}-\theta\Delta S_{s\to r}^{o}\begin{matrix} , & & & [2] \end{matrix}$$

where R is the gas constant (1.9872 cal mol^-1^ K^-1^) and θ is the temperature in Kelvin. By fitting the absorbance -temperature data to a straight line with equation [3]:

$-\Delta H_{s\to r}^{o}$and$\Delta S_{s\to r}^{o}$where are the slope and the intercept, respectively (see Fig. S-1). Since it was assumed that enthalpy and entropy did not vary with temperature, these values can be used to calculate $K_{s\to r}$ at each temperature. $K_{s\to r}$can also be used for calculation of (transition between probe–target duplex (bt) and molecular beacon in the form of a hairpin (b _closed_) at each temperature. is a parameter for calculating probe binding specificity (1).

**Table S-1**- Thermodynamic properties of capture probe.

| *T_m_* | $\Delta H_{s\to r}^{o}$ | $\Delta S_{s\to r}^{o}$ |
| --- | --- | --- |
| 60℃ | 34.291 kcal/mol | 103.06 cal/mol.k |


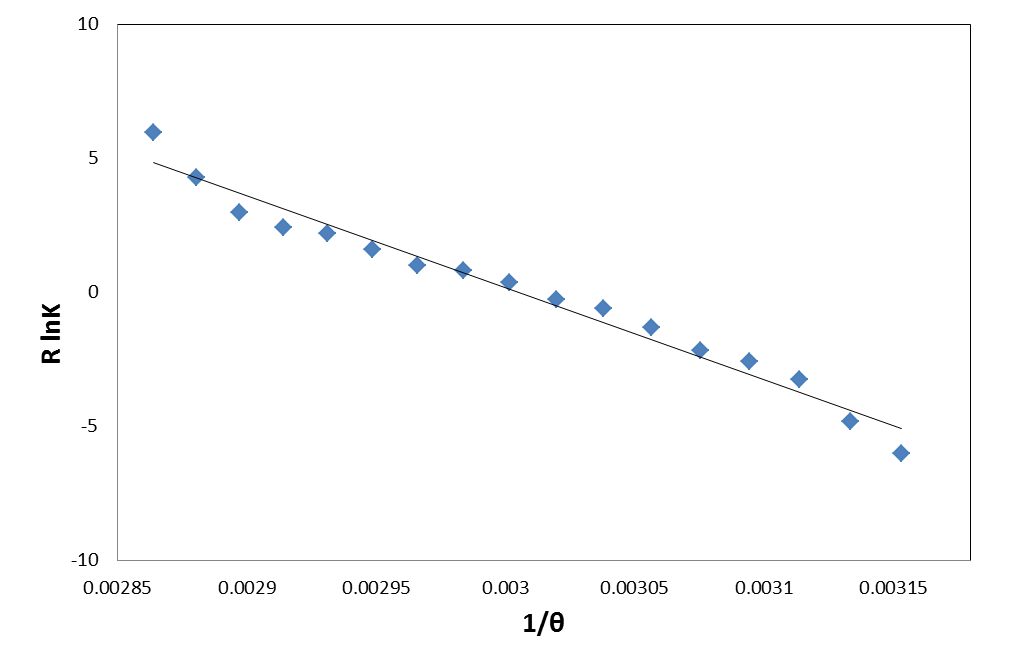


**Figure S-1-** Determination of thermodynamic parameters. The increase in absorbance that accompanies the melting of the capture probe was used to determine the thermodynamic parameters including enthalpy (slope of the fitted line) and entropy (y-intercept).

**2. Investigation of Stem-loop structure opening in the presence of target and non-target markers by Fluorescence spectroscopy and CD spectropolarimetry**

In order to better compare the samples and select the appropriate temperature for binding the miR-21 to capture, the data was plotted in separate charts (see Fig. S-2 and Fig. S-3 for Fluorescence assay and Fig. S-4 and Fig. S-5 for CD assay).


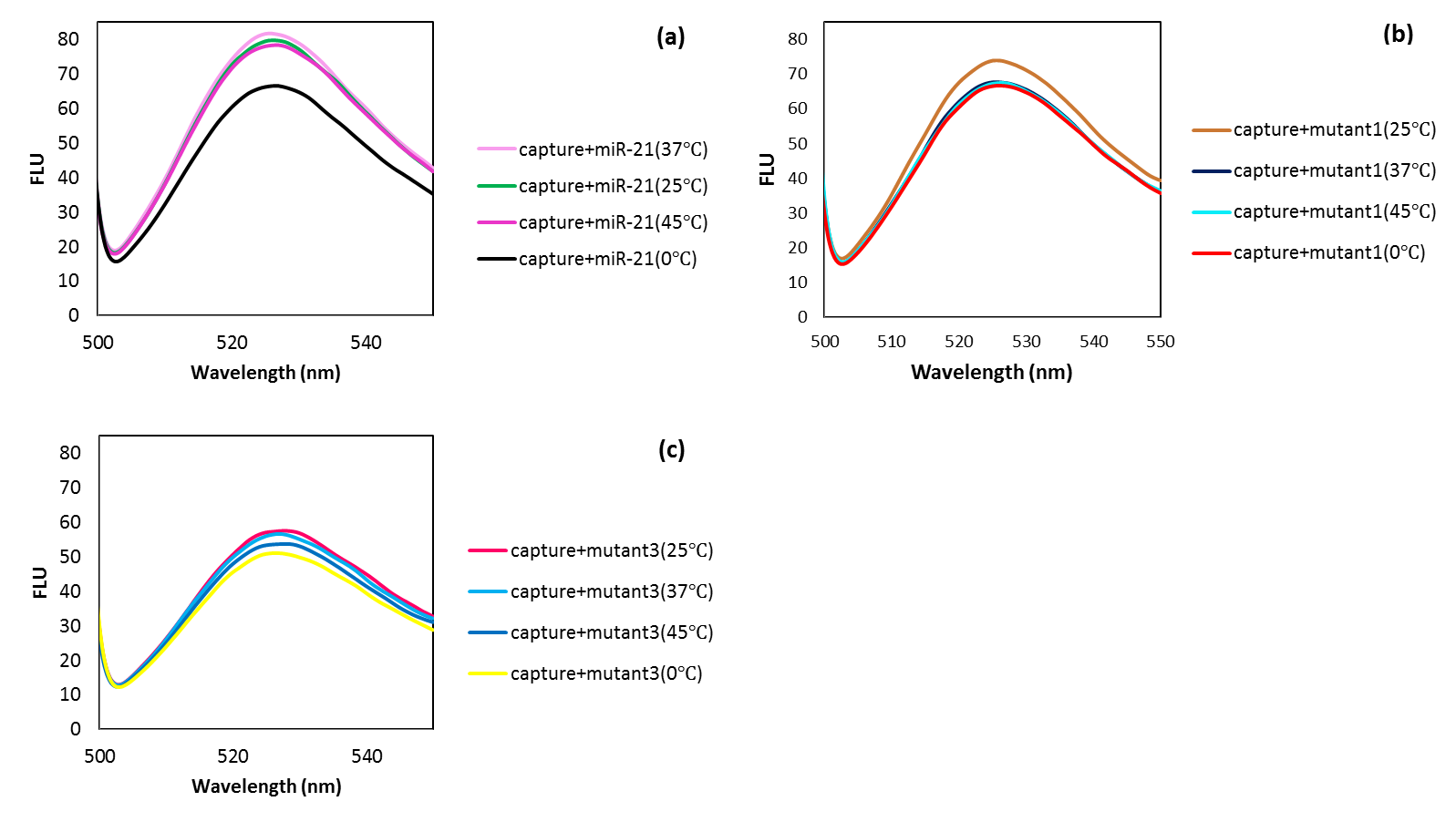


**Figure S-2**- Comparison of fluorescence intensity of capture-miR21 pair (a), capture-mutant1 pair (b) and capture-mutant3 pair (c) samples at 0, 25, 37 and 45 °C.


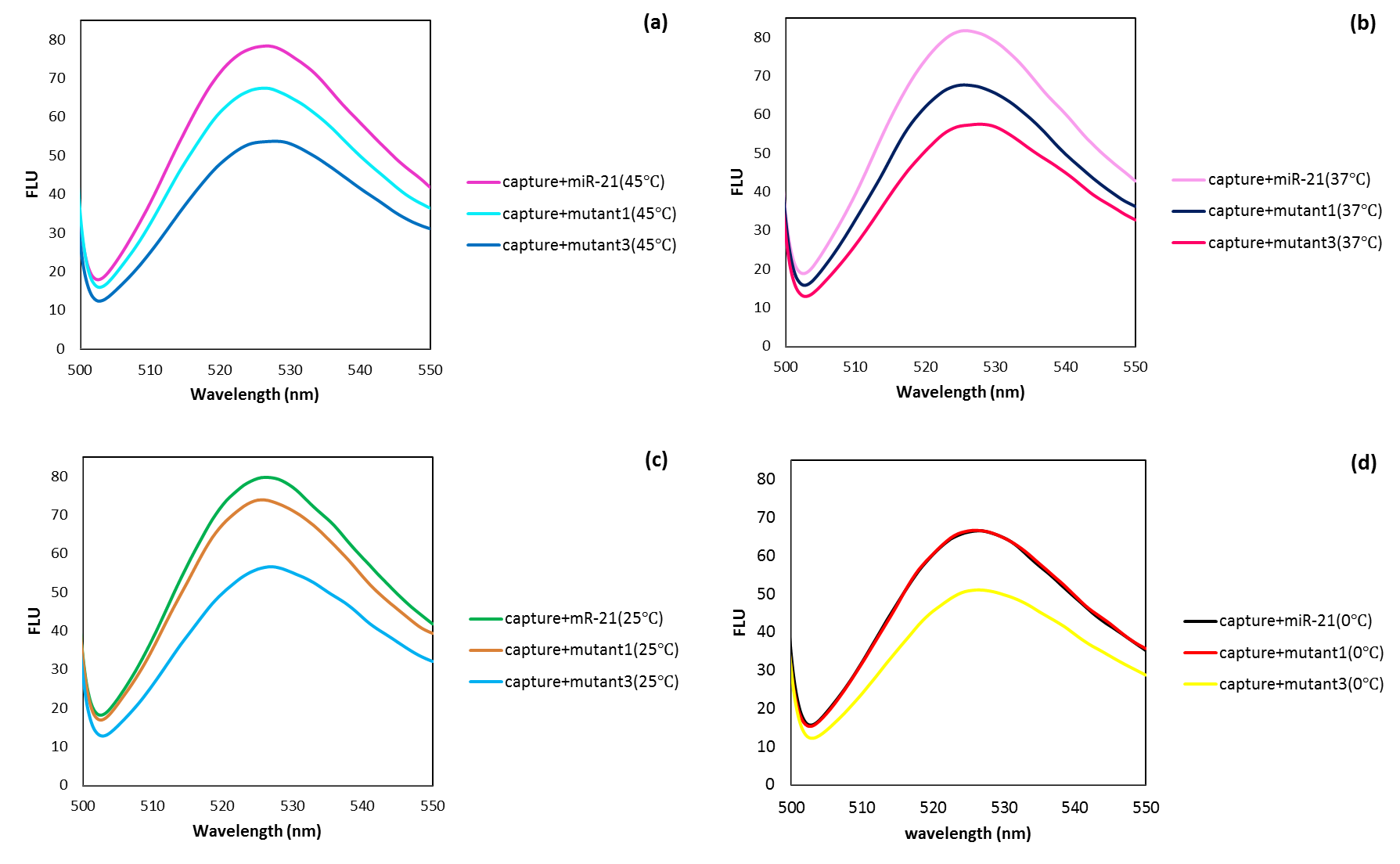


**Figure S-3-** Comparison of miR-21, mutant1 and mutant3 hybridization with capture probe at 45℃ (a); 37℃ (b); 25℃ (c) and 0℃ (d).

**
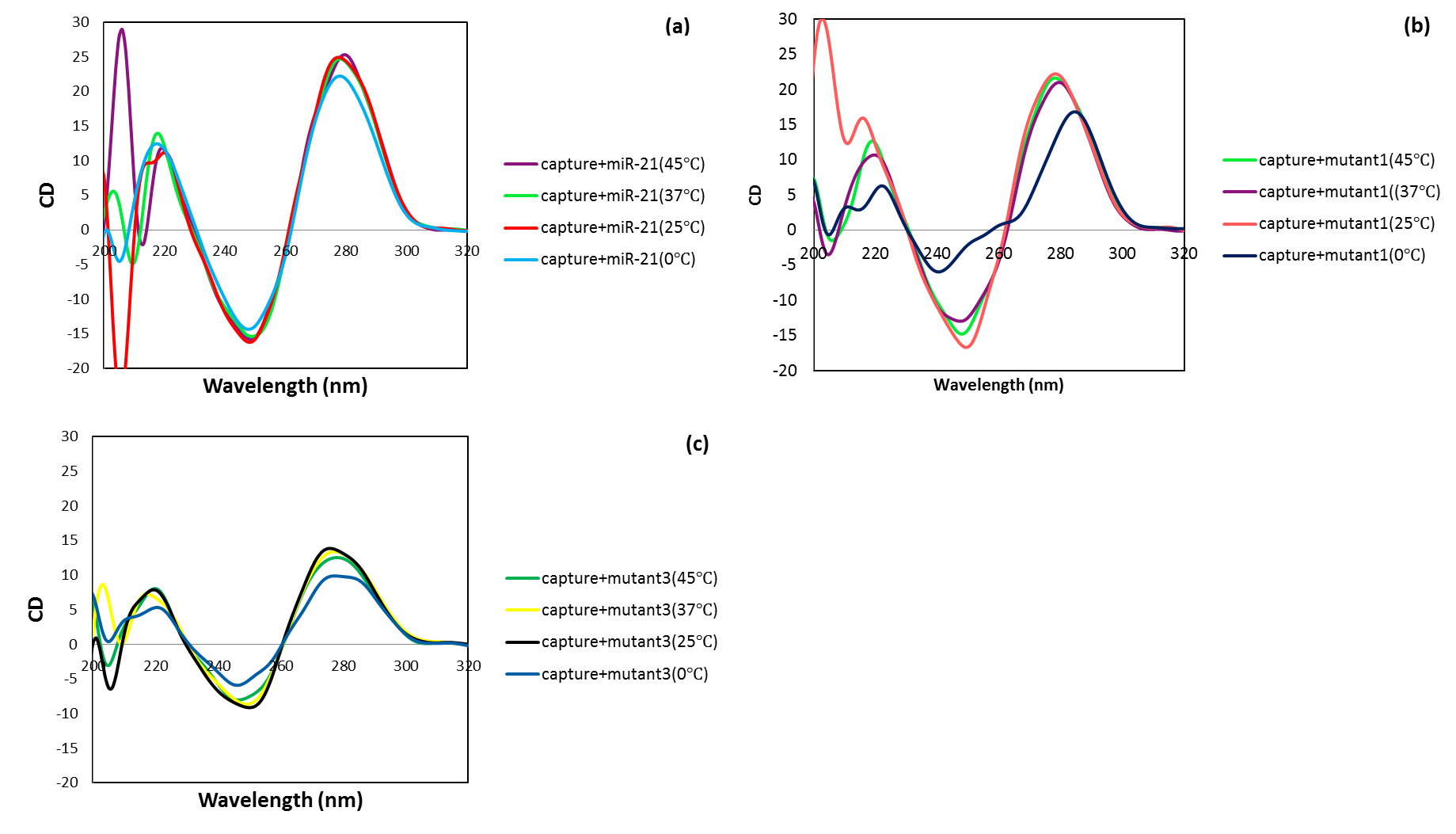
**

**Figure S-4-** Comparison of CD spectra of capture-miR21 pair (a), capture-mutant1 pair (b) and capture-mutant3 pair (c) samples under incubation conditions at different temperatures.

**
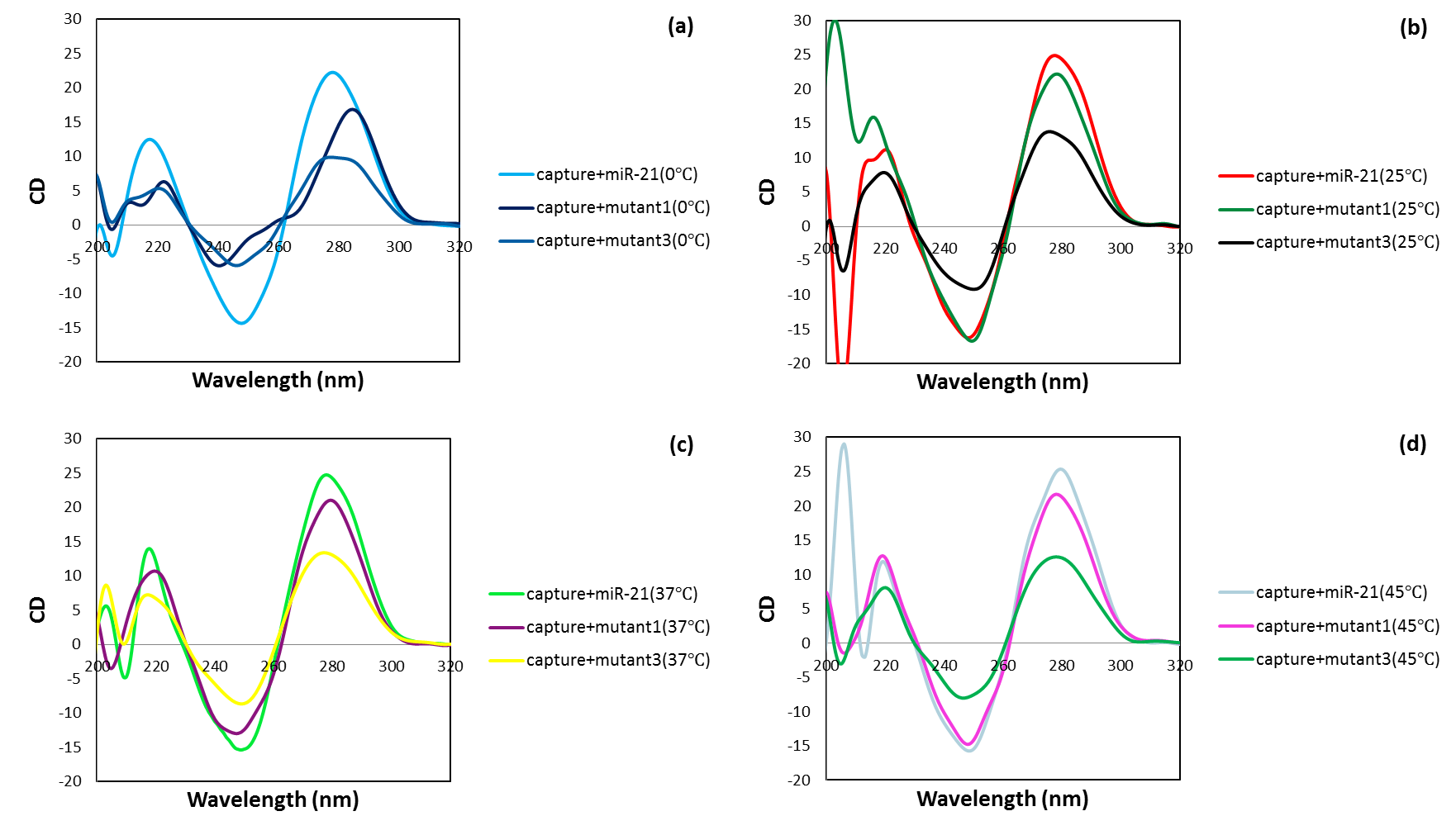
**

**Figure S-5-** Comparison of capture probe hybridization with miR-21, mutant1 and mutant3, depending on the incubation temperature

**3. Supporting Information References**

(1) Bonnet, G.; Tyagi, S.; Libchaber, A.; Kramer, F. R. Thermodynamic Basis of the Enhanced Specificity of Structured DNA Probes. *Proc. Natl. Acad. Sci. U. S. A.* **1999**, *96*, 6171–6176.
